# Effects of pyrolysis temperature, feedstock type and compaction on water retention of biochar amended soil

**DOI:** 10.1038/s41598-021-86701-5

**Published:** 2021-04-01

**Authors:** He Huang, Narala Gangadhara Reddy, Xilong Huang, Peinan Chen, Peiying Wang, Yuantian Zhang, Yuanxu Huang, Peng Lin, Ankit Garg

**Affiliations:** 1 Department of Civil and Environmental Engineering, Guangdong Engineering Center for Structure Safety and Health Monitoring, Shantou University, Guangdong, 515063 China; 2 Department of Civil Engineering, Kakatiya Institute of Technology and Science, Warangal, Telangana 506015 India; 3 L.N. Gumilyov Eurasian National University, Nur-Sultan, 010000 Kazakhstan

**Keywords:** Solid Earth sciences, Civil engineering

## Abstract

Recent studies on water retention behaviour of biochar amended soil rarely considers the effect of pyrolysis temperature and also feedstock type into account. It is well known that pyrolysis temperature and feedstock type influences the physical and chemical properties of biochar due to stagewise decomposition of structure and chemical bonds. Further, soil density, which is in a loose state (in agricultural applications) and dense (in geo-environmental engineering applications) can also influence water retention behaviour of biochar amended soils. The major objective of this study is to investigate the water retention properties of soil amended with three different biochars in both loose and dense state. The biochars, i.e. water hyacinth biochar (WHB), chicken manure biochar (CMB) and wood biochar (WB) were produced in-house at different pyrolysis temperature. After then, biochars at 5% and 10% (w/w%) were amended to the soil. Water retention behaviour (soil suction and gravimetric water content) was studied under drying and wetting cycle simulated by varying relative humidity (RH, 50–90%). Results show that 10% WHB produced at 300 °C were found to possess highest water retention. CMB is found to possess higher water retention than WB for 10% amendment ratio. In general, the addition of three biochars (at both 300 °C and 600 °C) at 10% (w/w) significantly improved the water retention at all suction ranges in both loose and dense compaction state as compared to that of the bare soil. The adsorption (wetting) and desorption (drying) capacity of biochar amended soils is constant at corresponding RH.

## Introduction

Growing environmental concerns and usage of natural sources have heightened the expedition of renewable sources. This has led to exploration of sustainable approaches including ecological restoration^1–4^. Biomass is an abundant and renewable resource. Current studies indicate^5–8^ that converting biomass waste into biomass oil, an alternative to fossil fuels, through thermochemical conversion technology is an attractive sustainable method. Among all biomass thermochemical conversion mothods, pyrolysis is a reliable approach to converts biomass into liquid bio-oil and solid biochar. Biochars (carbonaceous) are regarded as sustainable stabilizers because they are derived from pyrolytic biomass waste (i.e., plants, organic waste materials and animal waste)^9–11^. The obtained biochar is highly porous in nature with a high specific surface area and an abundance of hydrophilic groups. Biochars are widely used for soil amelioration, ecological restoration, waste management, engineered liner material and water treatment^12–14^.

A wide variety of biochars are produced from different feedstock such as invasive weeds (*Eichhornia crassipes, Prosopis juliflora*), animal and plant-based feedstock (chicken manure, pig manure, sawdust, peanut shell, straw waste; leaf waste)^15–19^. The biochar response to pyrolysis temperature degradation is different due to their inherent biopolymers and chemical composition of feedstock type^19,20^. Animal biochars are mostly constituted of animal protein such as gelatin, collagen, and polysaccharides (cellulose, starch and carbohydrates)^17,21^. Plant-based are mostly constitute of cellulose, hemicellulose, and lignin with a definite structure^11,22^. Kloss et al. and Chen et al.^15,19^ reported that various feedstock biochars produced under the same pyrolysis conditions have different properties. In addition, the pyrolysis process can also affect product properties. Fast pyrolysis is generally considered to be an efficient and feasible way to convert biomass into bio-oil^5–8^. On the contrary, the moderate heating rate during slow pyrolysis generally leads to the breakdown of weaker bonds while tending to retain stronger bonds^8^. Hence, such a rearrangement reaction promotes the structural stability of solid biochar.

On the other hand, the amendment of biochar and degree of compaction varies based on the application (i.e., agriculture or geo-engineering)^23^. The biochar amended soils are loosely compacted in agriculture applications (60–70% degree of compaction) while, the geo-engineered man-made structures such as embankments and landfill covers are typically compacted at higher density (85–95% degree of compaction)^24–26^. Available literature shows that the degree of compaction (loose and dense) affects the engineering properties of biochar amended soil^10,23,24^. The sorption and desorption properties of biochar amended soil mainly depend on available pore spaces in the soil matrix, which can be controlled by the compaction state (loose and dense). Moreover, these properties also depend on the feedstock type, and pyrolysis temperature^27–30^.

From a practical viewpoint, in addition to the above, temperature and humidity are the main factors that affect retention property usually denoted as soil water characteristic curve (SWCC). In general, the engineered landfills and slopes experience sorption and desorption, throughout the service period depending on environmental conditions^30^. For example, the minimum humidity in Inner Mongolia region of China and Rajasthan state of India (dry areas) experiences significant variation in summer (45–50%) and winter (i.e. > 80%). Monitoring of relative humidity can give an indication of soil suction near surface at different time intervals^26,31^. However, in the field study, it is difficult to interpret the fundamental behaviour of biochar amended soil due to natural variations in humidity and temperature as well as heterogeneity in soil. For understanding fundamental behaviour (sorption and desorption) of biochar amended soil, the humidity-controlled chamber tests are necessary for indirectly controlling suction^26,30,32,33^ in homogenous compacted samples. Based on the thermodynamic relationship, for a known temperature and relative humidity for each time interval, the total suction of the sample can be calculated by Kelvin equation using Eq. (1)^34^.

1 $$\psi_{t} = - \frac{RT}{{vM}}\ln \left( {\frac{RH}{{100}}} \right)$$

where $\psi_{t}$ is the total suction; *R* is the universal gas constant (8.314 J mol^−1^ K^−1^); *T* is the absolute temperature (K); RH is the relative humidity (%); ν is the specific volume of water (m^3^/kg); and *M* is the molecular mass of water vapour (18.02 kg/kmol).

Though, use of biochars as a sustainable amendment is widely explored, there are rarely any studies focussing on effect of relative humidity, feedstock type, pyrolysis temperature, and degree of compaction (i.e., dense and loose soil) on sorption, desorption behaviour of biochar amended soils. It can be hypothesized that alteration in physio-chemical properties of biochar due to variation in feedstock and pyrolysis temperature could affect water retention capacity of soil. Besides, compaction of soil (loose or dense) could also influence pore filling effect of biochar, that may ultimately affect water retention capacity. The influence of these variations (i.e., feedstock, pyrolysis temperature and compaction) on water retention can be useful in the selection of appropriate biochar (feedstock and temperature) for applications in agriculture (loose soil) or geo-engineered infrastructure (compacted condition).

The major objective of this study is to investigate the soil water retention properties of three in-house produced biochars (wood, chicken manure, and water hyacinth) at two pyrolysis temperatures (300 and 600 °C) and at different density (i.e., loose and dense states). Three biochars were amended in soil at 5% and 10% (w/w%). Change in the mass of biochar amended soil samples were monitored regularly to deduce gravimetric (i.e., mass) water content. The soil parameters such as mass water content and deduced soil suction (i.e., using Eq. 1) were monitored for seven weeks by varying the relative humidity at a fixed temperature of 30 °C.

## Material and methods

### In-house production of biochars at different pyrolysis temperatures

**Figure 1:**
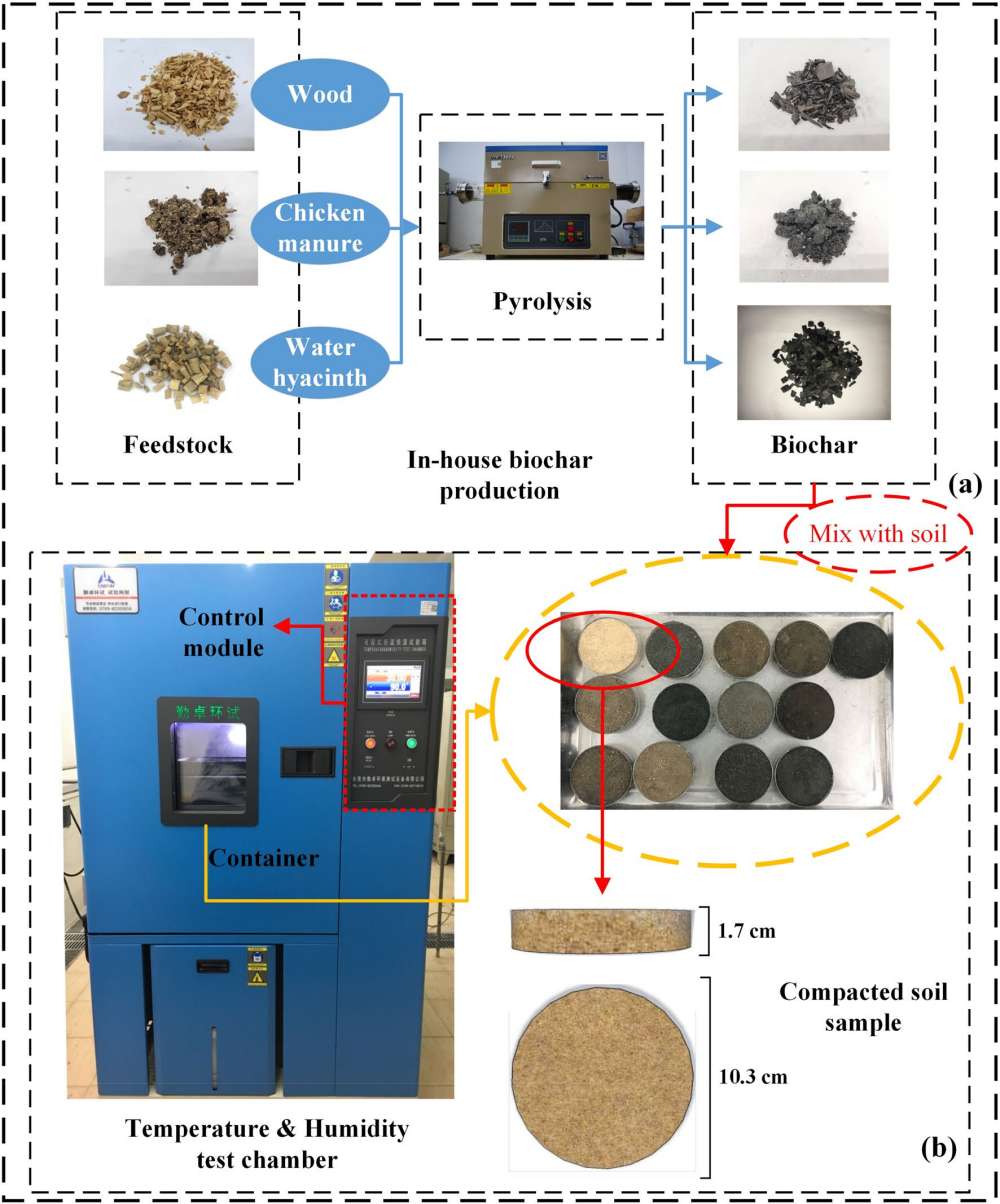
(**a**) In-house biochar production; (**b**) environmental test chamber and samples.

In total, three types of feedstocks were used to produce biochars in this study (as shown in Fig. 1a). Three waste materials include wood chips (i.e., rich in lignin), invasive plant water hyacinth (i.e., rich in cellulose) and chicken manure (i.e., partially digested organic matter). Feedstocks were preliminarily treated by air-drying for removing free moisture followed by fragmenting them into small pieces (10–20 mm). This was done to ensure complete pyrolysis of feedstocks in the furnace. Biochars were produced in a pyrolysis furnace (see Fig. 1a) under two different temperatures (i.e., 300 °C and 600 °C). Previous studies^8,35^ suggested that the breakdown of hemicellulose, cellulose, and lignin occur stagewise at temperatures approximately 195–255 °C, 235–345 °C, and 275–500 °C, respectively. Thus, two kinds of biochar can be obtained at pyrolysis temperatures of 300 °C and 600 °C by incomplete pyrolysis and almost complete pyrolysis, respectively. Pyrolysis process can be further interpreted by comparing the properties of biochar produced under different conditions (i.e., 300 °C and 600 °C). Slow pyrolysis process was selected to enhance the biochar yield. A heating rate of 10 °C /min was adopted till it reaches final equilibrium temperature (300 °C or 600 °C). Total pyrolysis period includes a 0.5 or 1 h heating period and 3 h residence period^7,8,28,36^. The produced biochars from pyrolysis were pulverized and passed through 2 mm sieve for further testing.

### Microstructural characterization studies of biochar

**Figure 2:**
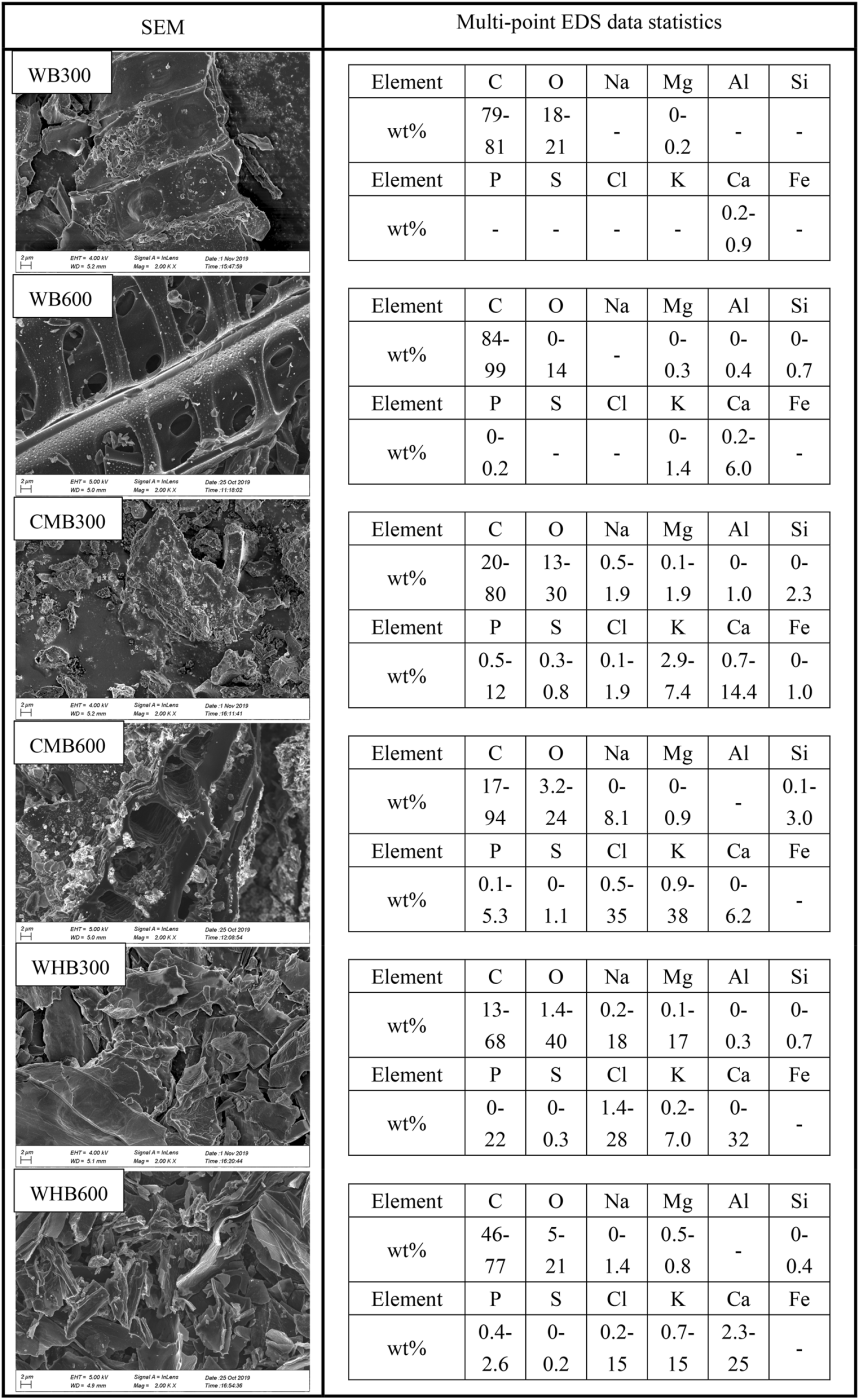
SEM and EDS analysis for produced biochar samples.

**Figure 3:**
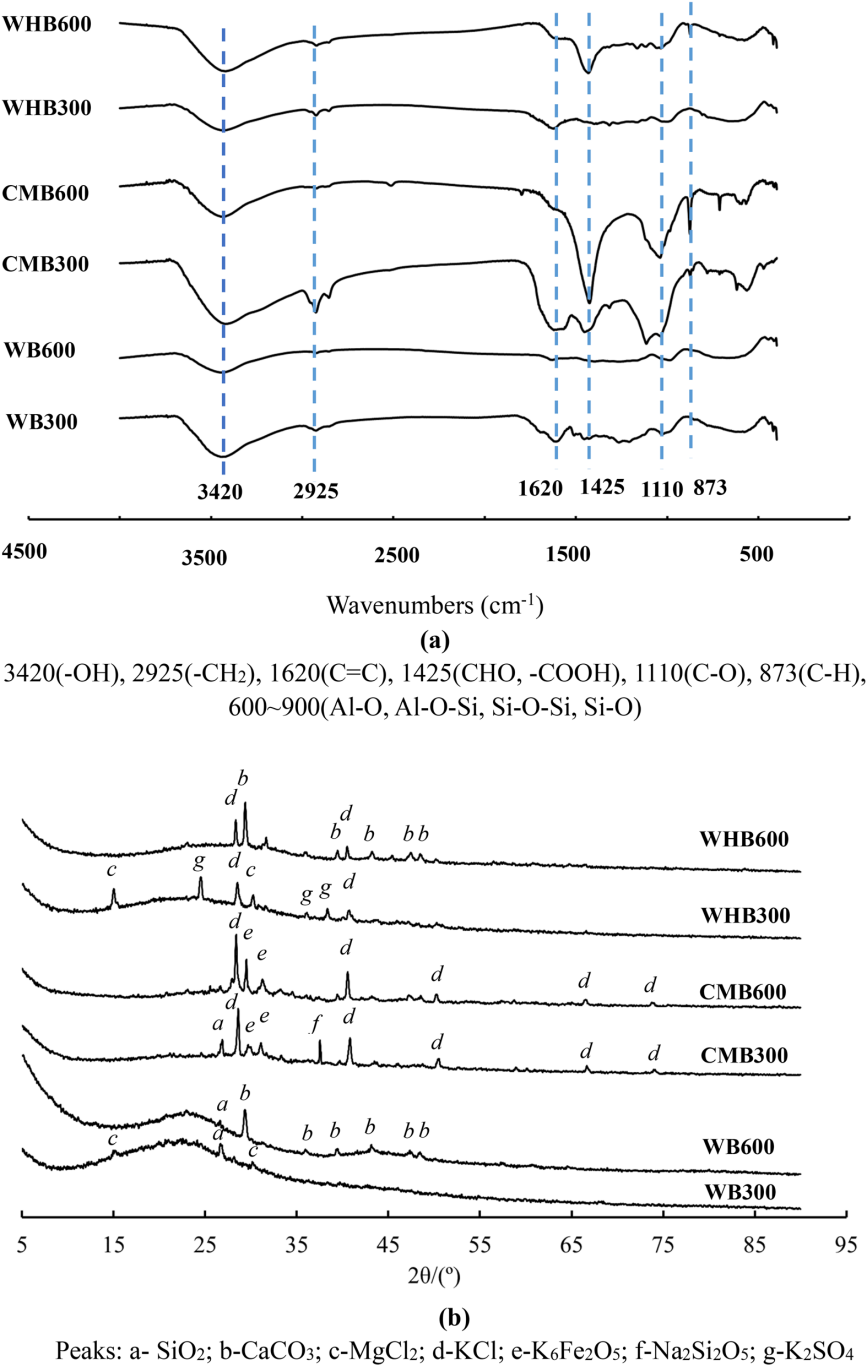
(**a**) FTIR analysis of biochar samples. (**b**) XRD analysis of biochar samples.

**Figure 4:**
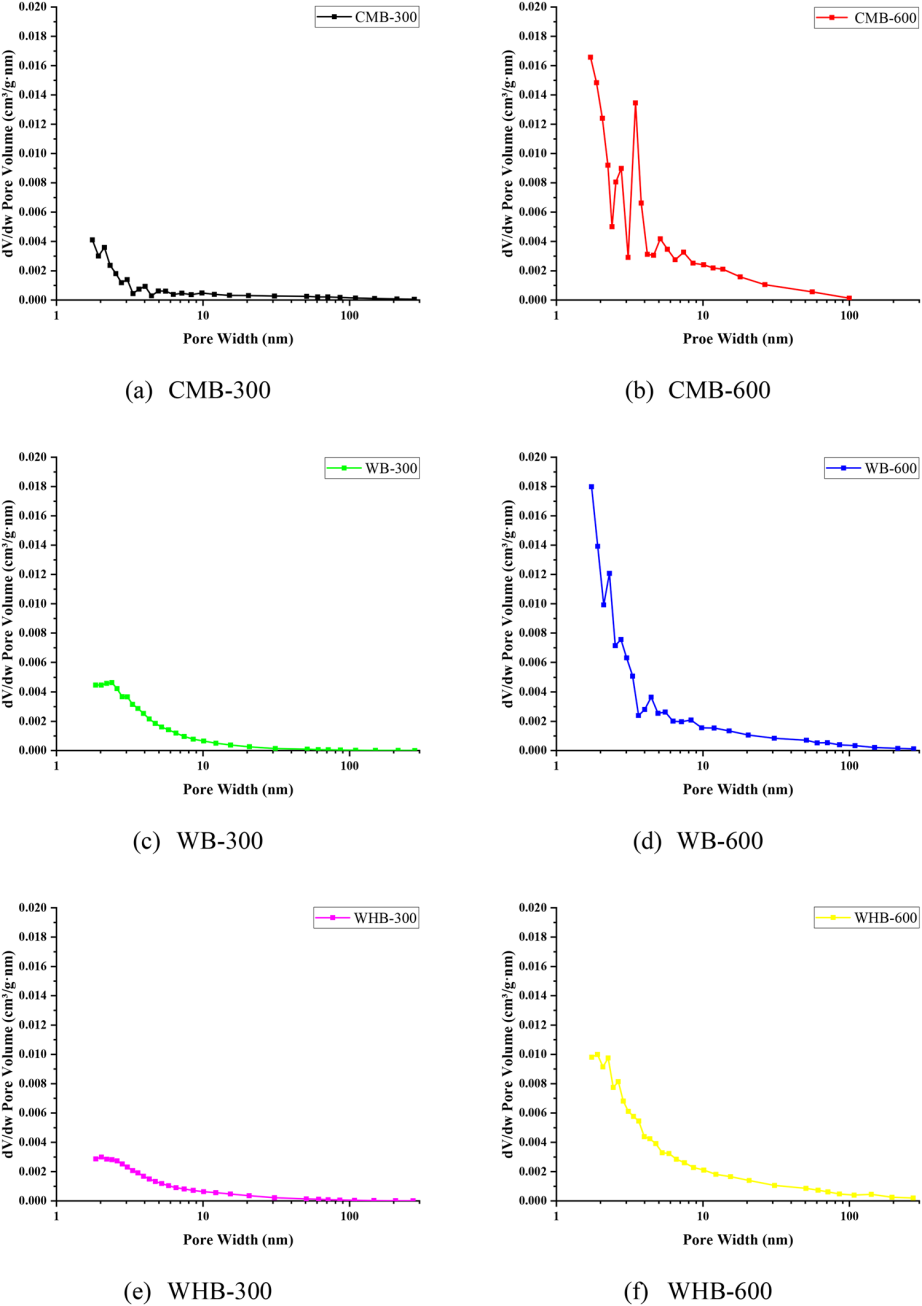
The pore size distribution of biochars.

Microstructural characterization was conducted for interpretation of the properties of biochars. Scanning electron microscope (SEM) studies were performed using Gemini 300 FE-SEM (Zeiss, Germany). For this purpose, biochar were precoated with platinum to provide electrical conductivity. Elemental analysis was performed with EDS technique, which is equipped with SEM. Organic element compositions (C, H, O, N, S) were measured using elemental analyzer (VARIO EL cube; Elementar, Germany). 50 mg of biochar passed through sieve No. 35 (0.5 mm) was used for this analysis. The C, H, N and S contents were measured in helium atmospheres. The O content was then measured in a helium–hydrogen atmosphere. Fourier transform infrared spectroscopy (FTIR) tests on various biochars were conducted with the help of a spectrophotometer (Thermo Nicolet Corporation, USA). The spectrum ranges of 4000–400 cm^−1^ with a resolution of 5 cm^−1^ were recorded. X-ray diffraction analysis was performed using the D8 advance X-ray powder diffraction device (manufacturer: Bruker, USA). Various biochar samples were scanned for reflections with 2θ ranging from 5 to 90° and with a step size of 0.03°. Scanning was done at a time interval of 0.5 s for each step. The specific surface area (SSA) and pore size distribution were quantified using ASAP 2020 (make, Micromeritics, USA) BET analyser based on N_2_ adsorption–desorption method. The purity of nitrogen used in this study is about 99%. Before testing, sample was kept in degasser for about 2 h at a temperature of 200 °C. The adsorption and desorption of isotherms were established at relative pressure (*p*/*p*_o_) intervals of 0.075–1. The properties of biochars are summarized in Tables 1 and 2. The microstructural analysis of produced biochars are shown in Figs. 2 and 3. The pore size distribution of all biochars are shown in Fig. 4.

**Table 1:** Specific surface area, pore width and pore volume of In-house produced biochar samples.

	WB300	WB600	CMB300	CMB600	WHB300	WHB600
Specific surface area (m^2^/g)	19.8	73.1	19.3	75.3	15.0	62.9
Mean pore width (nm)	6.14	10.87	10.87	16.03	8.68	13.81
Pore volume (cm^3^/g)	0.030	0.136	0.048	0.140	0.032	0.177
Yield (%)	45.0–48.3	23.7–28.0	60.7–61.5	46.7–49.1	41.3–44.0	24.9–27.2

**Table 2:** Elemental composition of in-house produced biochar samples.

Elemental composition	C (w/w %)	H (w/w %)	O (w/w %)	N (w/w %)	S (w/w %)	Other
WB300	66.40	4.66	20.88	0.12	0.00	7.94
WB600	83.29	2.14	6.22	0.22	0.00	8.13
CMB300	32.46	2.85	21.40	2.33	0.52	40.44
CMB600	37.60	0.82	19.91	0.92	0.45	40.30
WHB300	43.82	3.34	23.94	2.14	0.13	26.63
WHB600	59.10	1.63	15.39	1.53	0.00	22.35

### Preparation of soil-biochar samples

The soil (granite eluvium) used in this study was collected from the mountain of Shantou city, which is located near southern coastal region of China. The geotechnical properties of the soil are summarized in Table 3. The soil is categorized as silty sand with a group sample of SM as per ASTM D2487^37^. As shown in Table 3, the soil contains about 40% coarse grains (> 2.36 mm) and 14% of silt and clay. The soil was passed through 2.36 mm sieve (No. 8) to reduce any influence of coarser particles on the test results. The impact of particle size is more prominent on relatively smaller soil samples. The maximum dry density (MDD) and optimum moisture content (OMC) of soil were found to be 15.1 kN/m^3^ and 18.4%, respectively.

**Table 3:** Soil properties.

Properties	Standard	Soil
**Particle-size distribution (mm)**	ASTM D 422	
10.0–12.5		0.35
4.75–10.0		12.5
2.36–4.75		27.3
1.18–2.36		16.7
0.60–1.18		11.9
0.30–0.60		7.07
0.15–0.30		5.76
0.075–0.15		4.32
0–0.075		14.1
**Atterberg limits**	ASTM D 4318	
Liquid limit (LL/%)		28.8
Plastic limit (PL/%)		24.6
Plastic index (PI/%)		4.2
MDD (kN/m^3^)	ASTM D 698	15.1
OMC (%)	ASTM D 698	18.4
Specific gravity	ASTM D 854	2.67
Specific surface area (m^2^/g)	BET	12.7

**Figure 5:**
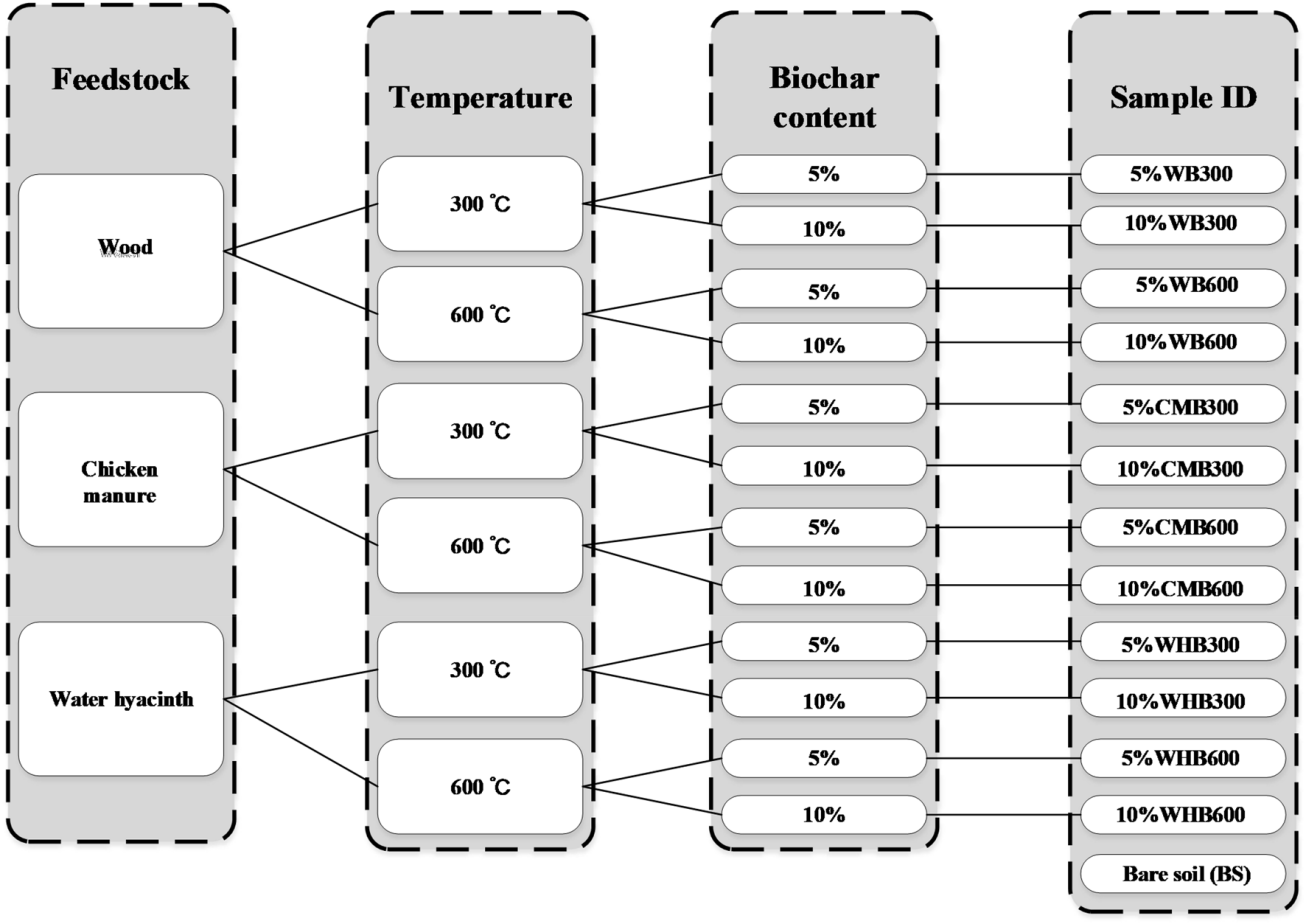
Biochar production conditions and sample grouping.

Bare soil was amended with biochar at an amendment rate of 5% and 10% (w/w%). The experimental groups were named accordingly (as shown in Fig. 5). The biochar feedstock, pyrolysis temperature, and dosage were used to distinguish different samples. For example, 5%WB300 represents the sample of wood biochar produced at 300 °C mixed with soil at 5% by weight. All the mixed biochar-soil composites were compacted into Petri dishes with 10.3 cm in diameter and 1.7 cm in height. In order to achieve similar initial conditions, all samples were compacted into the same state by controlling the weight. Loose (70% degree of compaction) and dense (85% degree of compaction) soil states, that are commonly used for agriculture and geo-environmental engineering applications, respectively were adopted^10,24–26^. A certain amount of deionized water was used for sample preparation to achieve the desired compaction state. All compacted samples were placed in an oven at 105 °C for 24 h to achieve initial dry state. To understand the physical properties of samples, specific gravity tests were conducted. Sample porosity and theoretical water content were calculated. The properties of biochar amended soil samples are summarized in Table 4.

**Table 4:** Properties of samples.

Loose	Bare soil	WB300		WB600		CMB300		CMB600		WHB300		WHB600	
Biochar content (%)	0	5	10	5	10	5	10	5	10	5	10	5	10
Specific gravity	2.67	2.60	2.57	2.59	2.58	2.60	2.55	2.62	2.57	2.56	2.46	2.54	2.45
Mass of dry sample (g)	153.8	157.4	157.0	157.1	157.1	155.7	157.8	155.8	156.7	156.1	155.3	156.3	155.2
Sample volume (cm^3^)	133.5	133.5	133.5	133.5	133.5	133.5	133.5	133.5	133.5	133.5	133.5	133.5	133.5
Sample porosity (%)	56.8	54.7	54.3	54.6	54.3	55.1	53.6	55.4	54.3	54.3	52.6	54.0	52.6
Theoretical maximum moisture content (%)	49.2	46.4	46.2	46.4	46.1	47.2	45.4	47.5	46.2	46.4	45.2	46.1	45.2

Dense	Bare soil	WB300		WB600		CMB300		CMB600		WHB300		WHB600	
Biochar content (%)	0	5	10	5	10	5	10	5	10	5	10	5	10
Specific gravity	2.67	2.60	2.57	2.59	2.58	2.60	2.55	2.62	2.57	2.56	2.46	2.54	2.45
Mass of dry sample (g)	187.9	189.8	189.2	190.2	189.8	190.5	189.7	191.4	190.0	188.8	188.9	189.1	188.5
Sample volume (cm^3^)	133.5	133.5	133.5	133.5	133.5	133.5	133.5	133.5	133.5	133.5	133.5	133.5	133.5
Sample porosity (%)	47.2	45.4	44.9	45.0	44.8	45.0	44.3	45.2	44.5	44.7	42.4	44.3	42.4
Theoretical maximum moisture content (%)	33.5	31.9	31.7	31.6	31.5	31.5	31.2	31.5	31.3	31.6	29.9	31.3	30.0

### Test plan

All the dry samples were placed in the humidity test chamber at a fixed temperature, (refer to Fig. 1), The temperature and humidity chamber are divided into two parts—the test chamber container part and the control module. The container part of the test chamber has a transparent glass observation area through which samples can be observed during the test. The programmable control module enables the chamber to adjust temperatures in the range of − 40 to 150 °C and humidity in the range of 20–100%. In this study, the temperature (30 °C) was kept constant, whereas, relative humidity was adjusted as to control total suction (refer to Eq. 1).

The study was monitored for a period of 7 weeks by fixing the environmental chamber temperature at 30 °C. The RH was initially set at 90% to allow the sample to adsorb enough water in the test chamber. After the sample reaches an equilibrium state (constant weight), RH was then adjusted to 50%. The test chamber is relatively dry at this humidity, and as expected, the sample undergo significant drying. When the sample reaches the equilibrium state again, the RH was adjusted to 90%, but, with an increment of 10% RH at each step. Therefore, the experiment was divided into 6 periods according to different RH conditions. The moisture content of all samples was calculated by recording the change of sample mass continuously during the test. The moisture content of each sample at equilibrium is recorded as the maximum moisture content under the RH condition. The water adsorption behaviour of samples with time are shown in Fig. 7. Based on the known temperature and relative humidity in the environmental chamber, total suction of the sample was deduced using the Kelvin equation (Eq. 1).

### Statistical analysis

Based on the studies conducted by Bordoloi et al.^38^ and Ulyett et al.^39^, both the water content and the relevant data were analyzed with analysis of variance (ANOVA). Statistical significances were determined based on criteria (P < 0.05). ANOVA analyses the contribution of variation from different sources to the total variation. It is useful to determine the influence of controllable factors on the water retention behaviour of biochar amended soil. The results of the ANOVA are summarized in Table 5.

**Table 5:** Summary of ANOVA results including significance of factors.

Source	Partial SS	df	MS	F	P
Model	97.0640	7	13.8663	37.3000	0.0000
C%	16.8197	1	16.8197	45.2500	0.0000
H%	20.1261	1	20.1261	54.1400	0.0000
O%	14.6530	1	14.6530	39.4200	0.0000
Other%	0.0412	1	0.0412	0.1100	0.7397
SSA	20.5497	1	20.5497	55.2800	0.0000
BC%	10.2487	1	10.2487	27.5700	0.0000
RH	56.5078	1	56.5078	152.0200	0.0000
Residual	44.9785	121	0.3717		

Where *SS* sum of squares, *df* degree of freedom, *MS* mean of squares, *BC* biochar, *%* w/w%, *Other* other elements (except C, H, O) in biochar.

## Results and discussion

### Characteristics of in-house produced biochar samples

The properties of three in-house produced biochar samples at temperatures of 300 and 600 °C are summarized in Table 1. It can be observed that the biochar yield produced at 300 °C temperature is higher than the biochar produced at 600 °C irrespective of the type of feedstock type. The WHB has lower yield as compared to WB and CMB. WHB typically constitutes 35–45% cellulose, 25–45% hemicellulose, and 20–30% lignin. The biochar produced from *water hyacinth* consists of more organic materials or biopolymers^38–40^.

The SEM images (at the same magnification) of biochar samples are shown in Fig. 2. Visual inspection of photographs exemplifies the differences in irregular and distinct porous surfaces. These observations are consistent with all the biochar samples. However, the biochars produced at high pyrolysis temperature (i.e., WB600, CMB600, and WHB600) shows larger porosity than biochar samples produced at low pyrolysis temperature (Fig. 2). The SEM results of biochars confirm that the SSA and pore width are comparable for higher pyrolysis temperature samples (Table 1). The pore volumes are in the range of 0.03–0.48 cm^3^/g and 0.136–0.177 cm^3^/g for pyrolysis temperature of 300 and 600 °C respectively. Further, the pore width range of biochar samples shows 6.14–10.87 nm and 10.97–16.03 nm for a pyrolysis temperature corresponding to 300 and 600 °C, respectively. The SSA of CMB is high as compared to WB and WHB at a given temperature. The pore volume distribution shows that the pore width of all biochar is mostly concentrated in the range of less than 10 nm, as shown in Fig. 4. Moreover, the pore volume of biochar produced at a pyrolysis temperature of 600 °C is significantly higher than 300 °C. It is a known fact that the pyrolysis temperature plays a vital role in biochar properties. The study results are matching to the previous studies of Brown et al., Singh et al., and Yargicoglu et al.^21,41,42^ who has reported relatively more porous structure, pore-volume, and SSA at high pyrolysis temperature and reduced biochar yield.

The elemental analysis of in-house produced biochars was analysed by adopting EDS technique. The percentage of each element is shown in Fig. 2. In all the biochar samples, carbon is found to be significantly high as compared to other elemental compositions. Carbon values are ranging from 13 to 96%. Oxygen is the second major element, followed by a few other minor elements (Mg, Na, Cl, Ca, K). The bulk elemental composition performed by element analyzer is summarized in Table 2. The analysis shows that WB has relatively higher carbon content at 83.29% (WB600). CMB possess much lower carbon content (32.46%, CMB300), indicating a larger number of inorganic compounds in biochar.

In general, FTIR analysis shows the presence of functional groups such as O–H, C–H, C=C, C–H, C–O, and C–O–C in the sample. Figure 3a shows the FTIR spectrum of biochar samples. The wavenumber at 3420 cm^−1^ shows the OH group, which belongs to the adsorbed water present in the water. The OH group is common in all biochar types irrespective of pyrolysis temperature (300 or 600 °C). The absence of functional groups viz. carboxyl (–COO–) and hydroxyl (–OH) in biochars are due to higher pyrolysis temperature during biochar production^40,43^. It is clear from the FTIR analysis that temperature influences the functional groups^21^. The wavenumber 2925, 1620, 1425, 1110, and 873 ~ 900 cm^−1^ belongs to the alkyl/aliphatic C–H stretching, aromatic C–C ring stretching, C–H alkanes, C–O–C symmetric stretching and aromatic C–H groups respectively^20,40^. The occurrence of most of the phases in WHB is due to the degradation of cellulose, hemicellulose, and skeletal lignin, which are present in the raw material^43^. The present FTIR test results are similar to that of Li et al.^20^, who also investigated WHB characterization.

The XRD patterns are indicative of the crystalline and amorphous structure present in the material. Figure 3b shows the XRD spectrum of the biochar samples obtained from three waste materials at 300 and 600 °C. The broad peaks in XRD at 2θ values between 25° and 30° possibly attributed to crystallinity in the lattice of cellulose. The hemicellulose and lignin are both amorphous in nature^40^. The XRD studies reported by Shabban et al. and Wang et al.^44,45^ shows similar patterns of the study for wood-derived biochar samples at varying temperatures. It is also observed that at low temperature (i.e., 300 °C) the biochar has narrow peaks at 16° and 24°, which disappeared at 600 °C. This might be due to the presence of cellulose at low temperatures. The study of Shabban et al.^50^ worth mentioned here that the cellulose starts decomposing at 315 °C and may finally disappear at higher temperatures. The peaks in WB and WHB are observed until 52°, whereas CMB shows both peaks till 75°. The peaks at 2θ = 31°, 36°, 42°, 43°, and 52° belong Na, Si, Mg, Ca, and K, respectively. These are virtually amorphous phases and display distinctive characteristics.

### Physical properties of biochar amended soil

**Figure 6:**
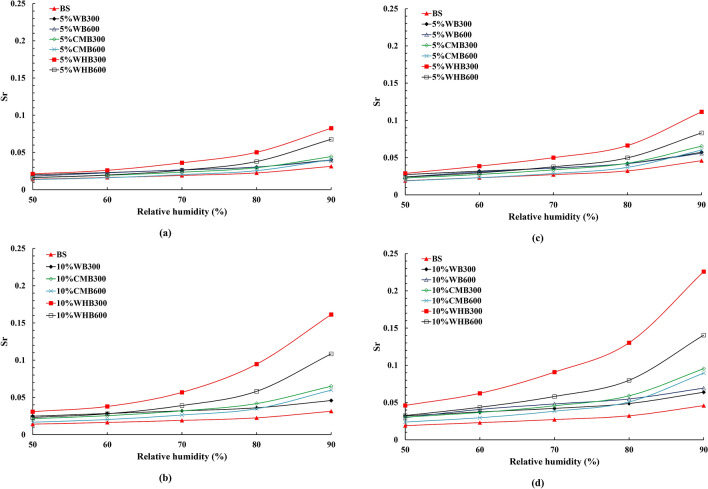
Saturation of (**a**,**b**) loose and (**c**,**d**) dense samples under different humidity conditions.

Table 4 summarizes the physical properties of soils amended with biochars. The specific gravity of bare soil (Gs) is 2.67. The Gs of biochar amended soils is low compared to the bare soil. The Gs values are in the range of 2.54–2.62 and 2.45–2.58 for 5% and 10% biochar content, respectively, for all the biochar types. It is observed that the effect of pyrosis temperature on Gs values of biochar amended soils is trivial. The reduction in Gs values is mainly attributed to the fact that the replacement of soil of particles by low-density biochar particles. The biochar amended soils porosity, and the maximum theoretical moisture content (gravimetric) were calculated for both loose and dense conditions (Table 4). The porosity values are in the range of 52.5–56.8% and 42.4–47.2, whereas, the theoretical moisture content values are in the range of 45.2–49.2% and 30–33.5% for loose and dense conditions respectively. The bare soil has high porosity and theoretical moisture content compared to the biochar amended soil samples. This is mainly due to relatively high compaction energy applied during sample preparation^28^. Further, the degree of saturation (Sr) of all the biochar amended soil samples was calculated. Sr signifies the amount of water present in the sample by taking compaction density (i.e., void ratio) and biochar content into consideration. Figure 6a–d shows the relation between Sr vs. RH for loose and dense samples under constant temperature conditions for 5 and 10% biochar content. The loosely compacted samples (Fig. 6a,b) show lower Sr than the sample compacted at high density (refer Fig. 6a,d). The densely compacted samples relatively have a low volume of voids at a given volume; thus, it translates high Sr values^46,47^. The results demonstrate that an increase in Sr value is more in biochar amended soils as compared to bare soil under enhancement of RH.

### Water adsorption and desorption behaviour of biochar amended soil

**Figure 7:**
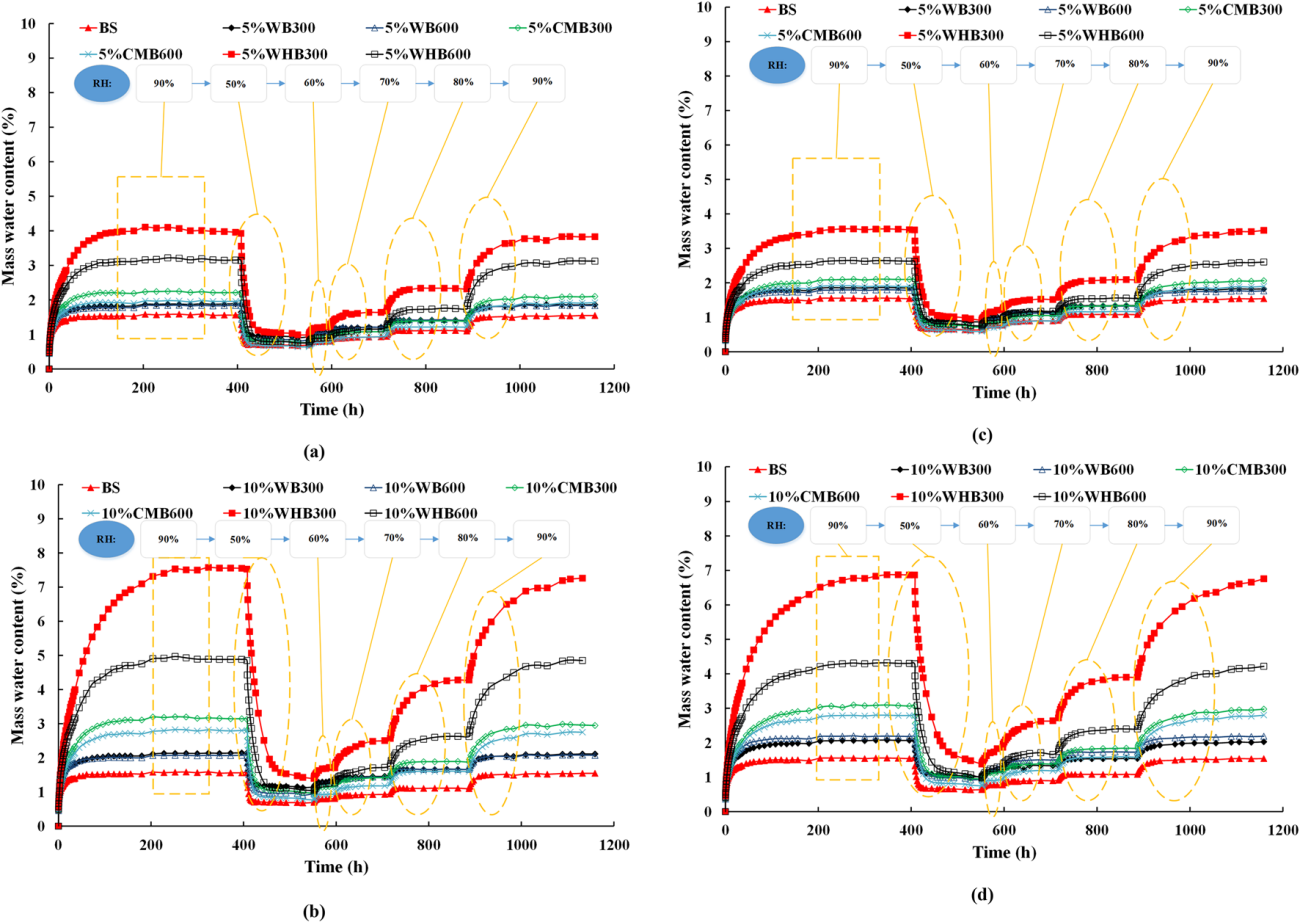
Water absorption behavior of different biochar amended (**a**,**b**) loose soil and (**c**,**d**) dense soil with time for various relative humidity conditions under 30 °C temperature condition.

The water adsorption and desorption curves of various biochar amended soil samples at loose and dense conditions are shown in Fig. 7. These tests are conducted for a period of seven weeks for different RH conditions at a constant temperature of 30 °C. The bare soil has a water content of around 1.56% at an RH of 90% (adsorption) and 0.73% at an RH of 50% (desorption), for loosely compacted sample. The high water adsorption at high RH is due to the available water in the test chamber^26^. The two extreme high and low RH values represent the humid (> 80%) and dry (50%) conditions, respectively.

The amendment of 5% and 10% biochar content increased the water adsorption capacity of the soil. The study of Mollinedo et al.^29^ shows that the application of different biochar types produced at varying pyrolysis temperature improved the water retention property of different soil types. These observations are in line with the present study. The water adsorption values are in the range of 1.78–2.18%, 1.89–3.14, and 2.6–7.55% for WB, CMB, and WHB, respectively, for 90% RH, which were produced at 300 and 600 °C. It was observed that the loosely compacted samples have high water adsorption capacity as compared to that of densely compacted samples. This is mainly due to the lower porosity of samples at high compaction density (see Table 4). The high amount of biochar (i.e., 10% biochar) has shown a positive effect on water adsorption properties of soil in both loose and dense states.

From Fig. 7, it can be observed that the time to reach an equilibrium of water adsorption is high at RH of 90%. For equilibrium, the maximum time is around 16 days (about 400 h). The is due to the presence of large number of small size pores in biochars that needs more time for equilibrium at a fixed RH as the samples were unsaturated; thus, the water adsorption process is slow^30,48^. The water desorption measurements of biochar (Fig. 7) show the gravimetric water contents were reduced drastically (from 0.7 to 1.2%) on the first day and attained equilibrium within two days for RH of 50%. Due to extremely low RH, biochar amended soil samples tends to loose retained water in the smaller pores. It can be noted that when the RH is increased by 10% in each increment, the time (3–6 days) for equilibrium is high. These test results demonstrate that the RH plays a vital role in both water adsorption and desorption of biochar amended soil samples.

**Figure 8:**
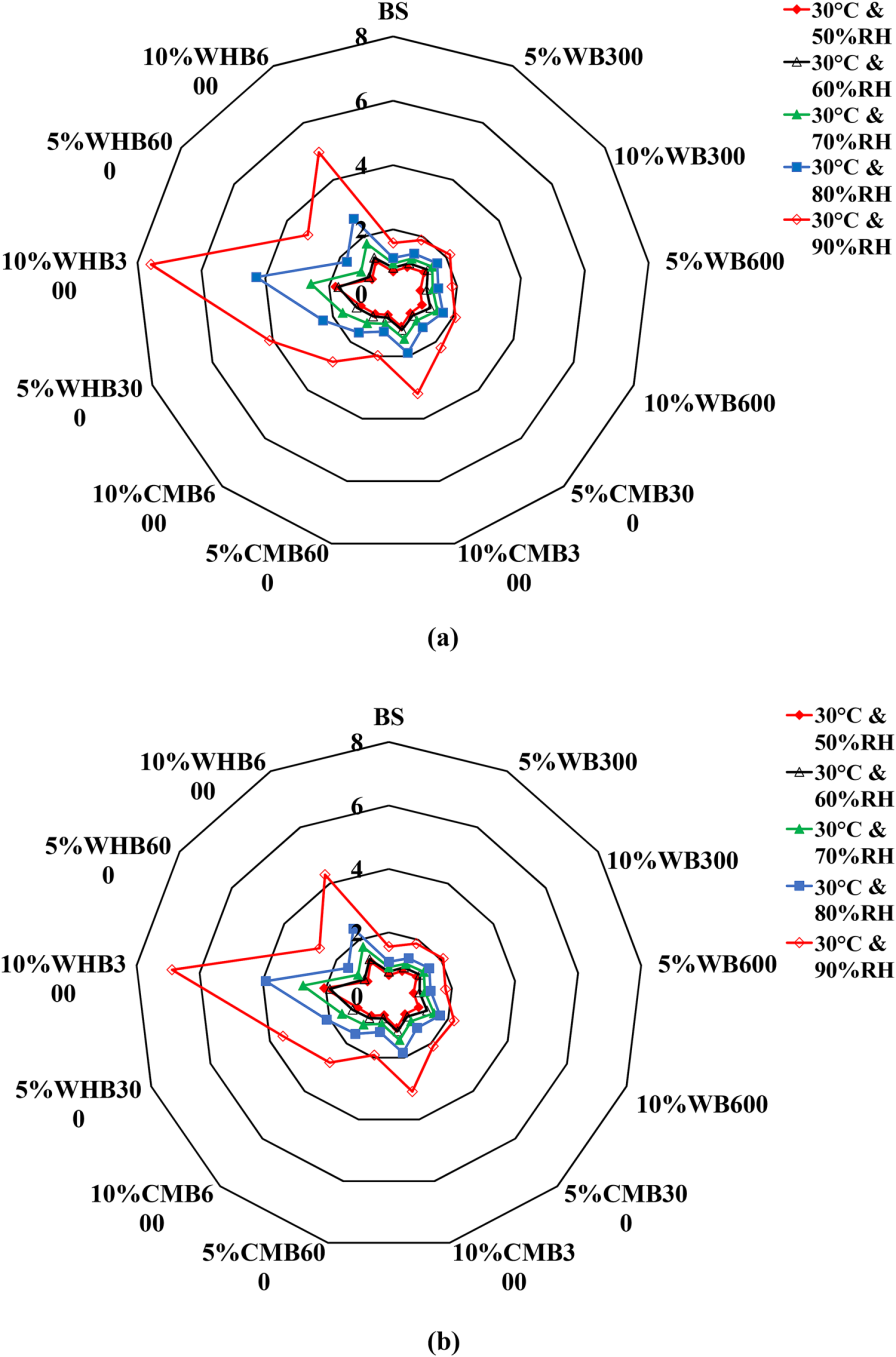
Maximum water content of (**a**) loose samples and (**b**) dense samples various relative humidity conditions under 30 °C temperature condition.

Figure 8 shows the maximum water content adsorbed by biochar amended soil samples under different compaction (i.e., loose and dense state) and pyrolysis temperature. The 10%WHB300 sample shows the high-water adsorption capacity than all other samples at both loose and dense conditions. Figure 8 demonstrated that the effect of biochar produced at higher pyrolysis temperature on water adsorption is not significant. This is despite the enhancement in specific surface area and porosity at higher pyrolysis temperatures. This is possibly due to the dissolution of organic matter and functional groups present in the biochar at 600 °C that may not be favourable for water adsorption^10,26^. Overall, the 10%WHB300 shows a better water adsorption capacity of 7.55% and 6.9% for loose and dense compacted biochar samples than other biochar samples (WB and CMB). This is likely due to the presence of favourable functional groups, especially hydrophilic groups (OH) with neutral (C–O) as a secondary group present in the WHB^22^. However, it should be noted that the current result is purely based on experimental conditions. The optimal content of biochar in the agricultural application will also depend on the specific plant type, which is not explored in this study^49^. Hence, systematic investigations are needed for the field application, including the engineering properties of biochar amended soil, considering the simultaneous influence on plant growth in the long term^25^.

### Soil water characteristics curves of biochar amended soils with relative humidity

**Figure 9:**
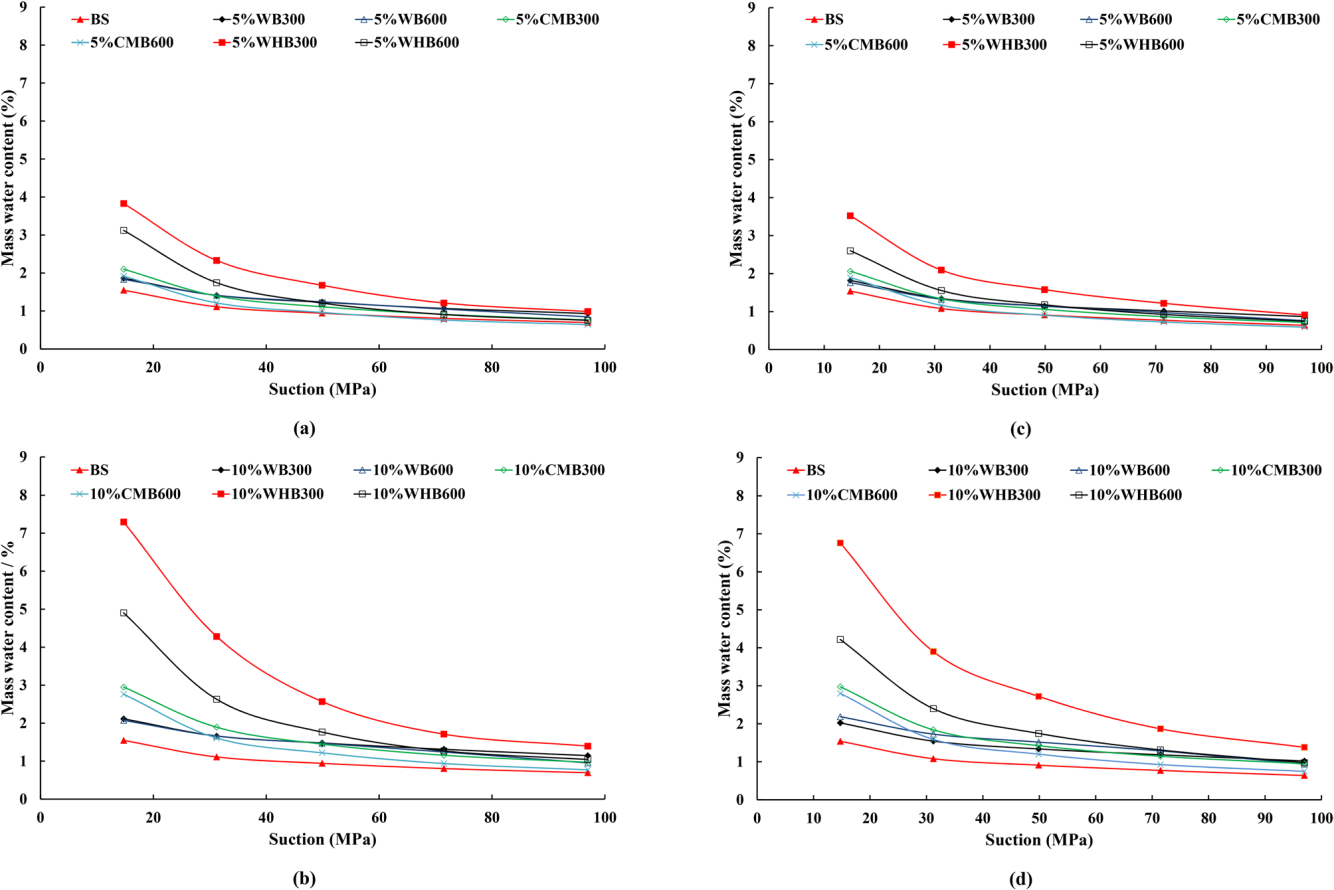
Moisture content of (**a**,**b**) loose and (**c**,**d**) dense samples various with suction.

Figure 9 shows the SWCC of bare soil and soil amended with various biochar produced from different feedstock and pyrolysis temperature compacted at loose (Fig. 9a,b) and dense (Fig. 9c,d) conditions. The deduced total suction varies between 14.7 and 96.9 MPa. The water content in bare and biochar amended soil is decreased with an increase in total suction for both loose and dense state (i.e. reduction of RH). For loose state, biochar amended samples significantly improved the soil water retention ability as compared to that in bare soil. However, an increase in soil density lowers the difference in water retention between biochar amended soils and bare soils. This is mainly attributed to presence of higher number of large pores in loosely compacted soil^50^. Pore filling effect of biochar is likely to have a higher impact on relatively loose soil as compared to densely compacted soil, which already possesses a larger number of smaller pores. The water retention ability of 5% amendment ratio is lower as compared to 10% in all biochar amended soil samples. This is because the larger biochar content enhances specific surface area and functional groups^38,45^. Moreover, the biochar amendment to soil increases the average void ratio of the mix. Eventually, the suction of the sample increases due to high capillary forces^13,25^.

With an increase in RH, mass water content in samples is increased. Since the suction (i.e., for a particular RH) is the controlling parameter in this study, the water retention of each sample is different, owing to variation in compaction state and biochar type (i.e., feedstock type and pyrolysis temperature). Such variation in water retention behaviour of biochar amended soils samples can be noticed in Figs. 8 and 9.

The increase in water retention of WHB produced (10%WHB300 and 10%WHB600) at loose and dense conditions is higher than the WB and CMB. WHB is highly hydrophilic in nature with exchangeable ions, thus retains more water that triggers electrical fields to improve short-range adsorption effects leading to increased suction^51,52^. On the other hand, WB and CMB have more inorganic content, which leads to lesser water retention (compared to WHB). Due to higher porosity, WB and CMB still retain more water than bare soil at the same total suction value^26^. The present results of the study are in line with the study of Bordoloi et al. and Wong et al.^26,38^ who has reported the addition of biochar improves the water retention capacity of the soil. However, effects of pyrolysis temperature and feedstock type on SWCC of biochar amended soils were not analysed in their study.

Table 6 summarizes the percentage of relative frequencies of the coefficient of variation (COV) for the data obtained from Fig. 9a–d. It can be observed from the statistical analysis that COV value falls below 30% for all experimental results. Moreover, 75% of data have COV of less than 15%, that shows the data is less variable for both loose and dense compacted samples. The COV’s demonstrate that the water retention results for biochar amended soils are reasonable and re-producible.

**Table 6:** Relative frequency of coefficient of variation (in %) for loose and dense compacted samples.

Bin interval	Loosely compacted samples		Densely compacted samples	
	300 °C	600 °C	300 °C	600 °C
< 5.0	31.3	50.0	37.5	25.0
5.1–10.0	18.8	25.0	25.0	18.8
10.1–15.0	31.3	12.5	12.5	25.0
15.1–20.0	6.3	12.5	12.5	0.0
20.1–25.0	6.3	0.0	6.3	25.0
25.1–30.0	6.3	0.0	6.3	6.3
> 30	0.0	0.0	0.0	0.0

### Mechanism of biochar effect on soil water characteristic curve

**Figure 10:**
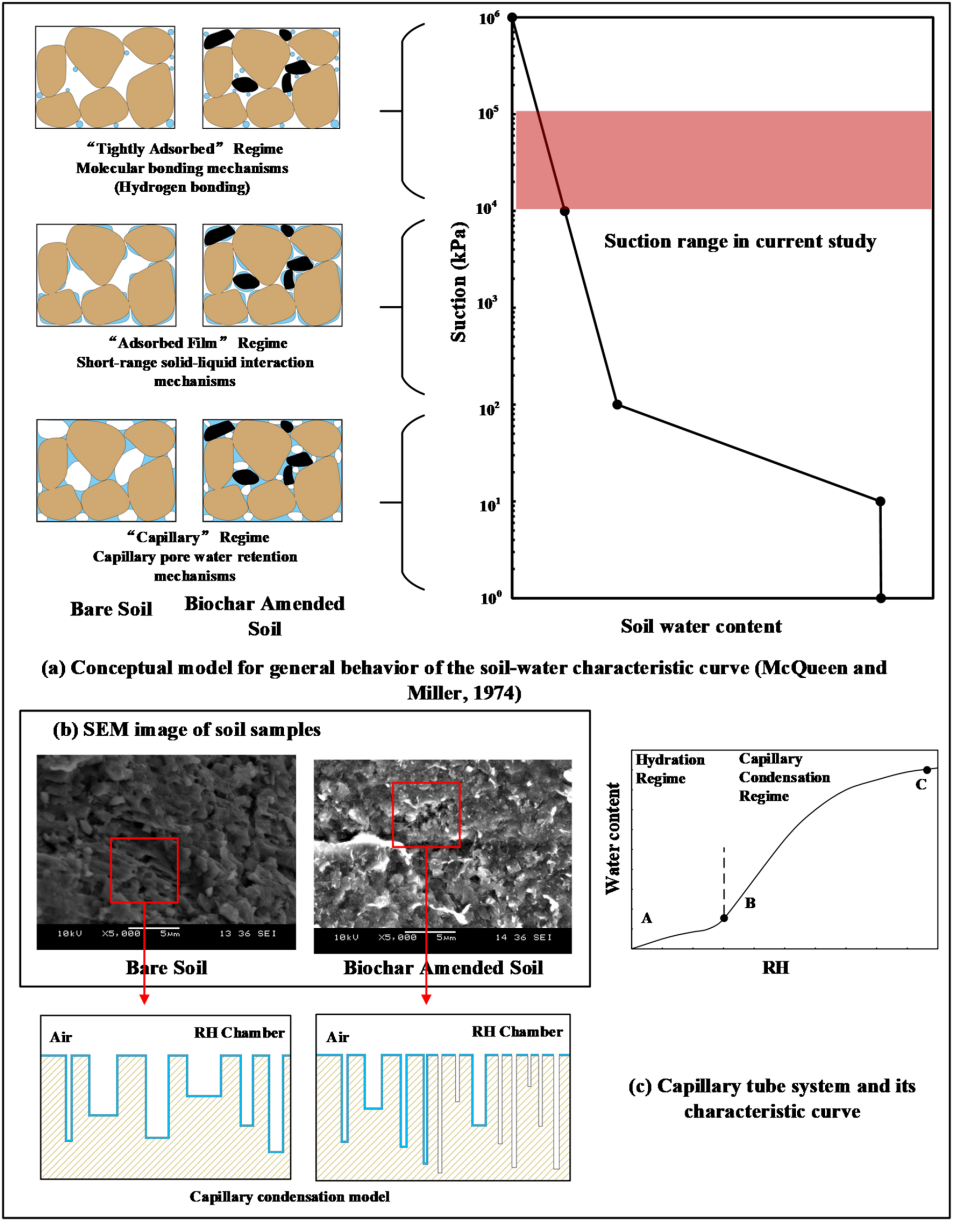
Conceptual models for water adsorption behaviour of the biochar amended soil.

SWCC generally depends on the properties of soil, organic material content, and mineralogy. The conceptual model of the general behaviour of SWCC proposed by McQueen and Miller^53^ suggests that the curve can be approximated as a combination of three straight lines in the logarithmic coordinate system from nearly zero to saturation state (see Fig. 10a). These line segments designated as (1) tightly adsorbed segment (about 10^6^–10^4^ kPa), (2) adsorbed film segment (about 10^4^–100 kPa), and (3) capillary segment (100 kPa to 0). In the current study, as shown in Fig. 10a, the suction range of the biochar amended soil samples in the controlled humidity chamber varies from 10^5^ to 10^4^ kPa. Within this suction range, pore water is primarily retained by molecular bonding with hydroxyl on the surfaces of the soil minerals and short-range solid–liquid interaction (e.g. polarization by electric fields, van der Waals attraction, and exchangeable cation hydration) in the form of thin films on the particle surfaces. The amount of adsorbed water is supposed to be related to surface area of soil particles, the valence of cations in minerals, and type of surface functional groups^54^. Higher water content in biochar amended soil than that of bare soil at the same suction (see Fig. 9) could be attributed to surface hydrophilic functional groups and larger surface area of biochar (refer to Figs. 2, 3, and Table 1). In addition, the changes in minerals, surface functional groups, and surface area caused by different types and contents of biochar also affects the amount of adsorbed water. In the present study, WHB and CMB have better water adsorption capacity than WB and BS. It was found that 10% amendment ratio provides better water sorption than 5% (Fig. 9).

In order to further interpret SWCC of biochar amended soils, the combined model of hydration and capillary condensation was visualized (refer to Fig. 10c; after Lu and Likos^54^). In the capillary tube system, the sizes and lengths of the capillary tubes are distributed according to the actual soil pore sizes. Figure 10b shows the SEM images (at a magnification of 5000) of biochar amended soil and bare soil. It can be seen from the figure that biochar amended soil has higher number of pores than that of bare soil. Further, it can be observed that laminar diaphragm divides the pores into smaller sizes. The corresponding hypothetical capillary tube system is shown in Fig. 10c. Such system indicates a lower pore size of biochar amended soil than bare soil.

In the combined model of hydration and capillary condensation, the water film is first formed in the capillary tube due to hydration. When the water film reaches a certain thickness, the surface effect of solid particle disappears, and capillary condensation becomes the dominant adsorption. The capillary tubes in the system are filled in sequence starting with smaller tubes. Therefore, the high adsorption ability of biochar amended soil is due to its small pores. In the characteristic curve of the capillary tube system, hydration plays a dominant role till point B (Fig. 10c). Capillary condensation plays a dominant role beyond point B (refer Fig. 10c). In general, biochar played a positive role in the whole adsorption process. In the hydration stage, the surface effects of the soil are enhanced due to presence of hydrophilic surface functional groups and higher surface area of biochar. During the capillary condensation stage, biochar changes pore size and hence, enhances soil water adsorption. Therefore, biochar has the potential as a soil amendment in improving soil water retention. This has been widely confirmed by previous research works^18,23,38^. Moreover, the decrease in pore size due to increased density can also enhance the capillary effect of the soil^23^. This can be observed from Fig. 6, where denser soil achieved higher saturation during the capillary condensation stage.

The ANOVA was conducted using Stata software for computing the significance of factors. Table 5 summarizes the significance of various factors in water content for biochar amended soil. It can be observed that C, H, O are significant based on the P value. Nevertheless, the content of other chemical elements are not significant. The original element composition of soil masked the influence of other elements (except C, H, O) in biochar. In addition, factors such as SSA, BC% and RH are also significant. The results further confirm the mechanism of biochar's influence on water retention and capillary condensation behaviour of soil discussed in this section.

### Discussion on the performance of biochar in water retention

As summarized in Table 7, many studies^38,39,51,52,55–58^ reported that biochar significantly increases the water retention ability of soil. Nevertheless, the effectiveness of biochar on water retention varies with biochar type, pyrolysis condition, biochar content, and soil properties. It is reasonable to summarize that the properties of biochar are affected by the feedstock and the pyrolysis process and hence, the soil water retention. In addition, most studies found that soil water retention increased with an increase in the biochar application amount. However, the study conducted by Abel et al.^55^ found that 5% maize biochar reduced soil water retention, while 1% and 2.5% had the opposite effect. Thus, biochar preparation should be optimized based on the needs of the utilization field^59^.

**Table 7:** The performance of biochar in water retention.

Study	Soil	Pyrolysis condition (°C)	Method	Improvement
Garg et al.^52^	Sand clay mixture	350–400	5% biochar from water hyacinth	6.5% increase in water retention
			10% biochar from water hyacinth	10.5% increase in water retention
Bordoloi et al.^38^	Sand clay mixture	300–350	15% biochar from water hyacinth	19.0% increase in water retention
Ulyett et al.^39^	Sandy loam soil (from the organic farm)	600	60 t/ha biochar from a deciduous mixed wood (sycamore, oak, beech and bird cherry)	5.3% increase in water retention
	Sandy loam soil (from the conventional farm)	600	60 t/ha biochar from a deciduous mixed wood (sycamore, oak, beech and bird cherry)	6.2% increase in water retention
Hardie et al.^51^	Dark brown–black sandy loam	550	47 Mg/ha from acacia whole tree green waste	9.9% increase in water retention
Abel et al.^55^	Sand	750	1% biochar from maize	10.3% increase in water retention
			2.5% biochar from maize	10.9% increase in water retention
			5% biochar from maize	5.8% reduction in water retention
Obia et al.^56^	Sand and loamy sand	350	1.7% biochar from maize cob	4.1% increase in water retention
			3.4% biochar from maize cob	7.7% increase in water retention
Sun et al.^57^	Clay	500	2% biochar from crop straw	1.4% increase in water retention
			4% biochar from crop straw	6.1% increase in water retention
			6% biochar from crop straw	18.4% increase in water retention
			6% biochar from woodchips and sawdust	8.7% increase in water retention
			6% biochar from Chinses medicine production sludge	6.8% increase in water retention
Bruun et al.^58^	Sandy soil	730	2% biochar from ground wheat straw	5.5% increase in water retention
			4% biochar from ground wheat straw	11.9% increase in water retention
		450–480	2% biochar from mixed hardwood (69% Norway Spruce, 19% other wood species)	3.6% increase in water retention
Present study	Sandy soil	300	5% biochar from water hyacinth	147% increase in water adsorption
			10% biochar from water hyacinth	371% increase in water adsorption
			5% biochar from chicken manure	35% increase in water adsorption
			10% biochar from chicken manure	90% increase in water adsorption
			5% biochar from wood	20% increase in water adsorption
			10% biochar from wood	36% increase in water adsorption
		600	5% biochar from water hyacinth	101% increase in water adsorption
			10% biochar from water hyacinth	216% increase in water adsorption
			5% biochar from chicken manure	24% increase in water adsorption
			10% biochar from chicken manure	78% increase in water adsorption
			5% biochar from wood	18% increase in water adsorption
			10% biochar from wood	34% increase in water adsorption

The current study presents a novel viewpoint by analysing water retention property (i.e., SWCC) for loose and compacted soils amended with biochars produced at different pyrolysis temperature and feedstock. Among all, 10% WHB300 is found to enhance soil water absorption by 371% (i.e., reference to bare soil). The improvement of soil water retention by biochar can be explained by defensing water loss under dry conditions and enhancing water adsorption under wet conditions.

### Cost analysis of biochars

The cost analysis equation (Eq. 2) for biochar production by previous studies^36,38,60^ is adopted for 1 ton of WB, CMB, and WHB.

2 $$C_{BC} = C_{FEEDSTOCK} + C_{PRODUCTION} + C_{EXTRA}$$

where, C_BC_ is the unit cost of biochar per ton, C_FEEDSTOCK_ is the cost of feedstock for per ton biochar, C_PRODUCTION_ is the cost of per ton biochar pyrolysis, C_EXTRA_ includes the cost per ton of transportation and so on. The prices are assigned as per cost standards of Shantou region of China.

The cost of feedstock in the current study are around 80, 60, and 100 USD/ton for wood, chicken manure, and water hyacinth, respectively. Considering the biochar yield ratio (Table 1), the C_FEEDSTOCK_ of WB300, WB600, CMB300, CMB600, WHB300, and WHB600 are around 177, 320, 100, 133, 250 and 400 USD/ton, respectively. The C_PRODUCTION_ is estimated as per laboratory conditions. The electricity consumption and other consumption required to produce per kg of biochar is around 0.6 USD, i.e. 600 USD/ton. However, in large-scale production, this cost will be effectively reduced due to increased capacity. The C_EXTRA_ considered transportation, and so on are around 50 USD/ton. Thus, the C_BC_ of per ton WB300, WB600, CMB300, CMB600, WHB300, and WHB600 are around 827, 970, 750, 783, 900, and 1050 USD, respectively. Such high prices are mainly due to the low production capacity of the laboratory pyrolysis furnace. The current price of biochar produced by factories in China are around 500–1000 USD/ton. Prices may fluctuate depending on the type of feedstock and the biochar yield. Biochar yield using Chicken manure and wood feedstock is relatively high, typically costs around 500 USD/ton. In the present research work, very small quantity of biochars were used for the determination of water adsorption and desorption properties. Therefore, it may not be reasonable to make a cost comparison for research use at smaller scale. Further, systematic studies are needed to determine the cost–benefit comparison of biochars produced from different feedstocks and pyrolysis temperature at large scale based on field application (agriculture farms or geo-engineered landfill or slopes).

## Conclusions

The study explored the effect of in-house produced biochars from different feedstock type and temperature at loose and dense states on water adsorption, desorption, and suction parameters of soil. The in-house produced biochar yield was between 28 and 61 wt% and surface area in the range of 15–19.8 m^2^/g and 62.9–75.3 m^2^/g. The study shows that RH has a definite effect on the water adsorption and desorption properties of biochar amended soils. Higher water retention was observed in 10%WHB300 and 10%WHB600 samples at both loose and dense states due to favourable functional groups (hydrophilic) and porosity of WHB biochar than other two biochars. Biochar amended soil retains more water at the lower suction range (i.e., RH of 90%) as compared to the bare soils. However, at higher suction, differences in water retention between bare soil and biochar amended soil is less significant. The water sorption and desorption behaviour of biochar amended soils is similar at a particular relative humidity. Since the experiments were conducted at higher suction range, water retention in pores are mainly dominated by intermolecular forces and short-range adsorption. This study suggests 10% biochar application rate (WHB > CMB > WB) has significantly improved the water retention capacity of soil. The results presented in this study indicated that the pyrolysis temperature, feedstock type, and density state need to be considered for selection of biochar as an amendment in cover material in landfill covers.

This study presents a novel viewpoint on water retention and capillary condensation behaviour of biochar amended soil. The results show that biochar can significantly improve the water absorption capacity of dry soil under wet conditions. This study complements the research on water retention of soil modified by biochar. Nevertheless, water retention ability of biochar amended soil under extreme climatic conditions (such as drought and freeze–thaw) is also worth exploring in future studies.

## Abbreviations

WHB: Water hyacinth biohar
CMB: Chicken manure biohar
WB: Wood biohar
BC: Biochar
BS: Bare soil
RH: Relative humidity
$$\psi_{t}$$: Total suction
R: Universal gas constant
T: Absolute temperature
v: Specific volume of water
M: Molecular mass of water vapour
SEM: Scanning electron microscope
FE-SEM: Field emission scanning electron microscopy
EDS: Energy dispersive spectrometer
FTIR: Fourier transform infrared spectroscopy
XRD: X-ray diffraction
SSA: Specific surface area
BET: Brunauer–Emmett–Teller
MDD: Maximum dry density
OMC: Optimum moisture content
Gs: Specific gravity
Sr: Degree of saturation
ANOVA: Analysis of variance
SWCC: Soil–water characteristic curve
COV: Coefficient of variation
C_BC_: Unit cost of biochar per ton
C_FEEDSTOCK_: Cost of feedstock for per ton biochar
C_PRODUCTION_: Cost of per ton biochar pyrolysis
C_EXTRA_: Cost per ton of transportation and so on
SS: Sum of squares
Df: Degree of freedom
MS: Mean of squares
